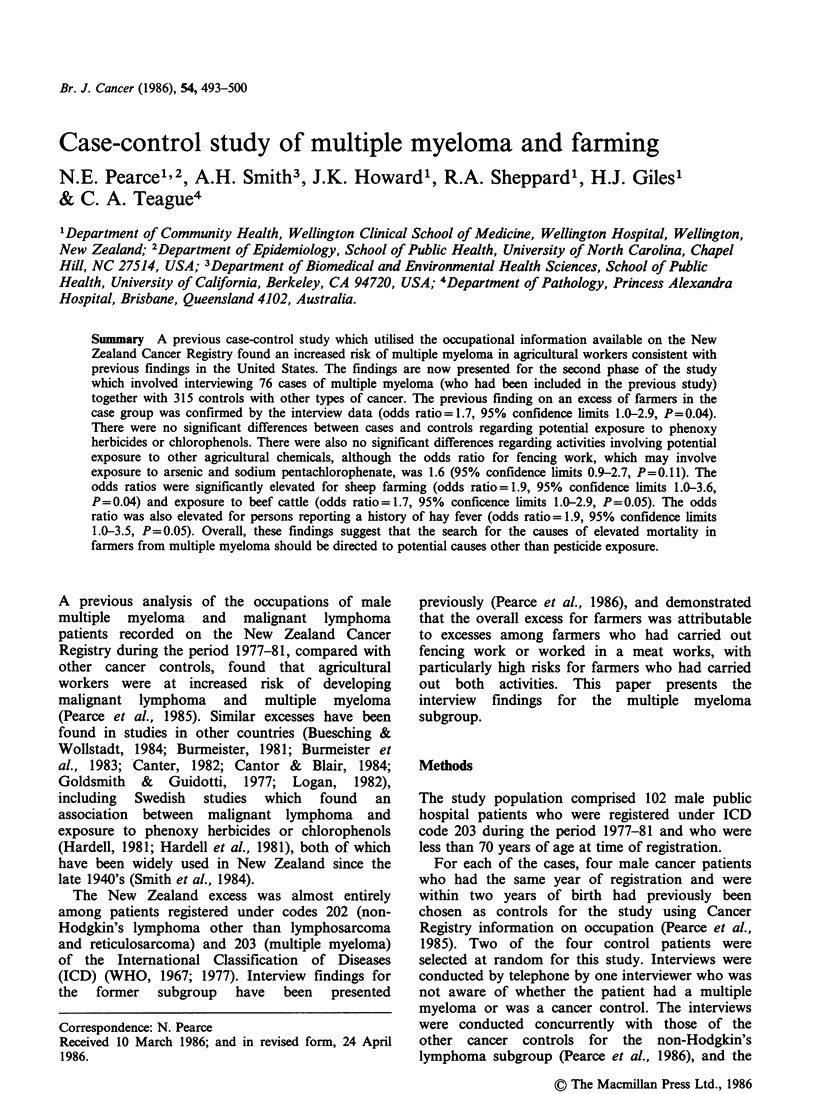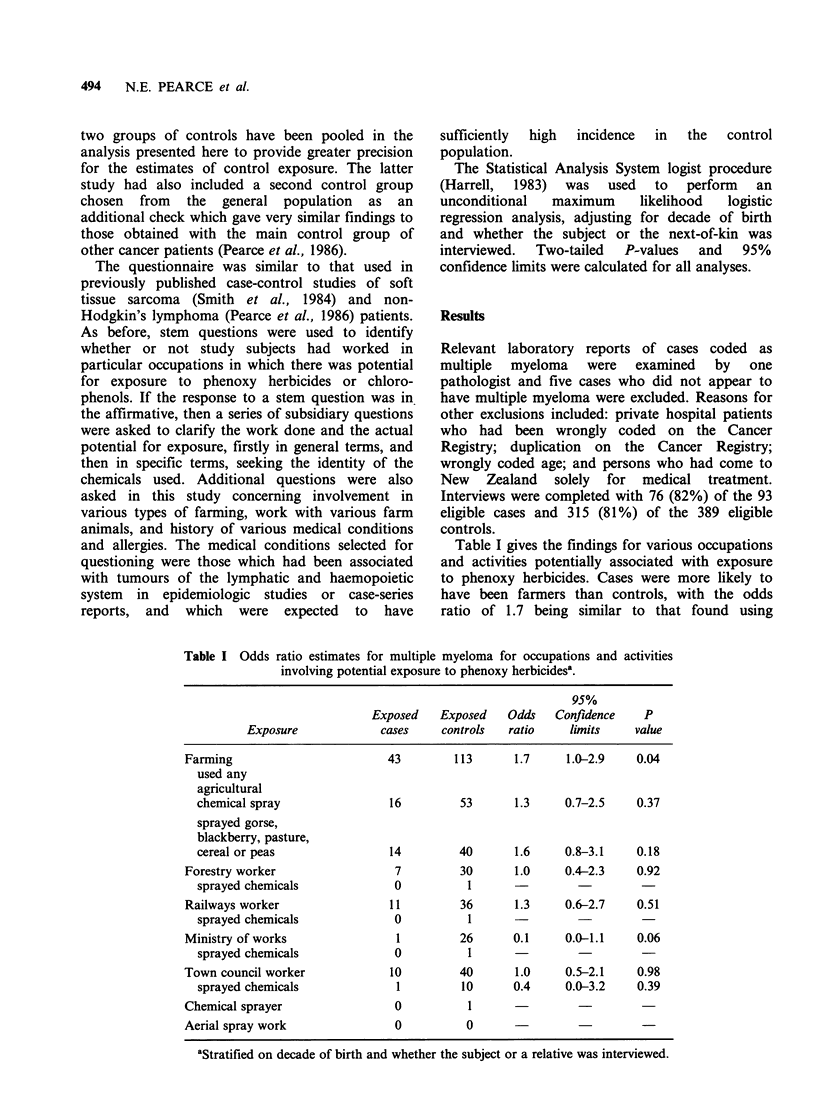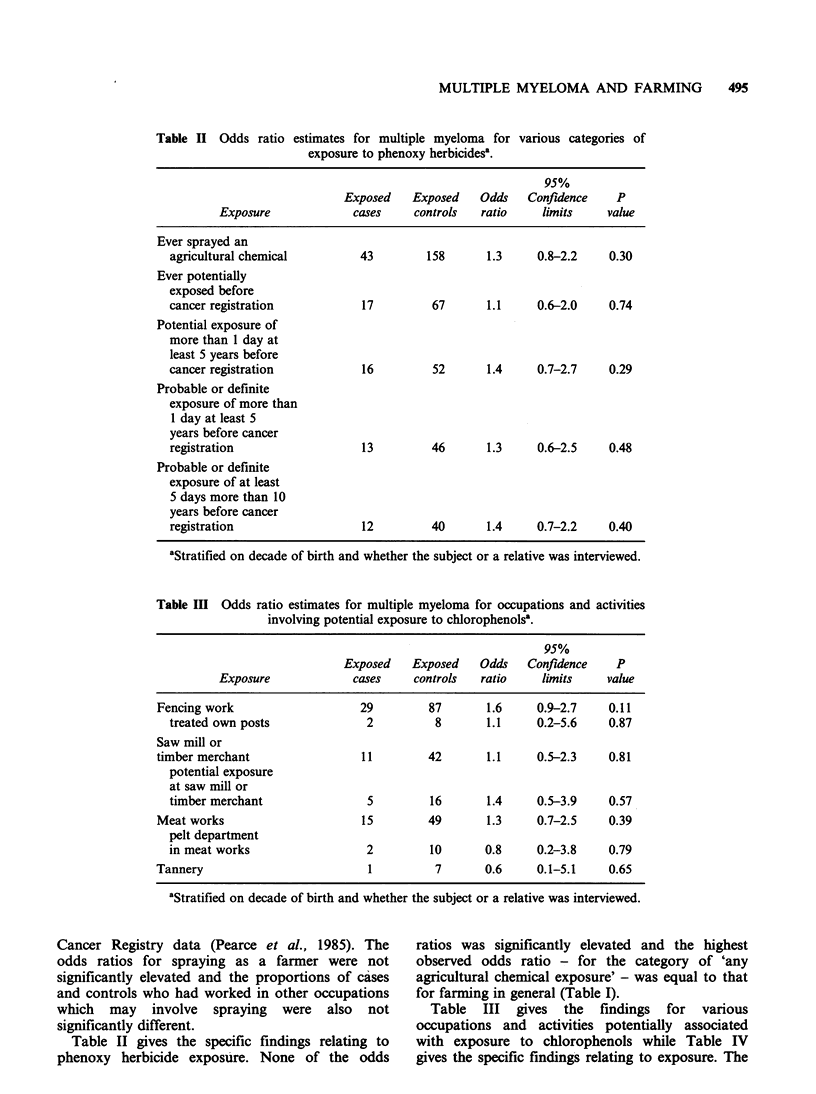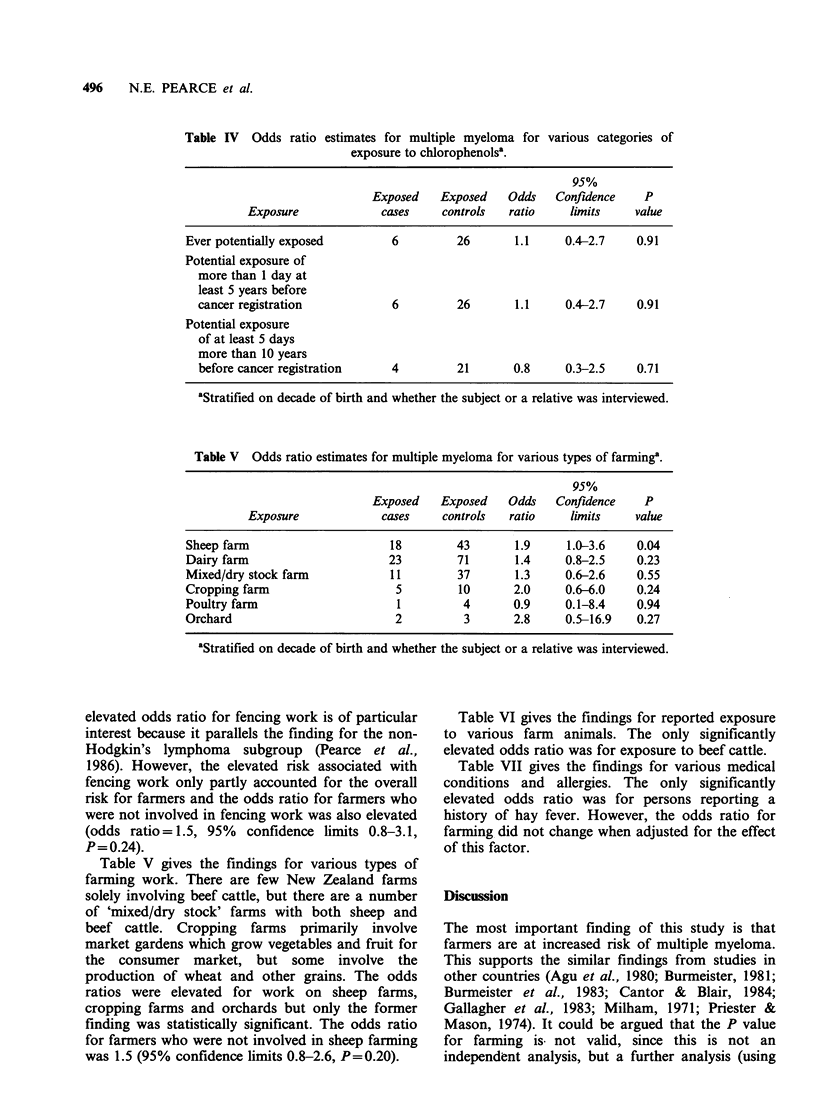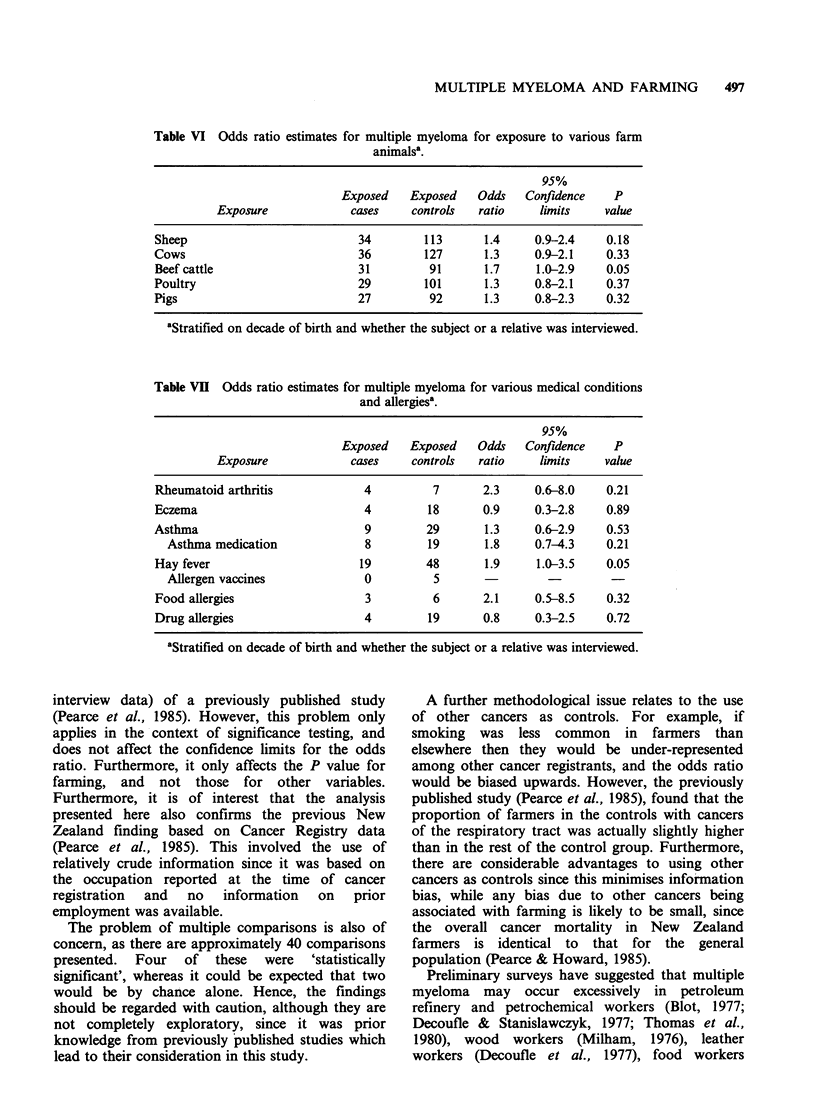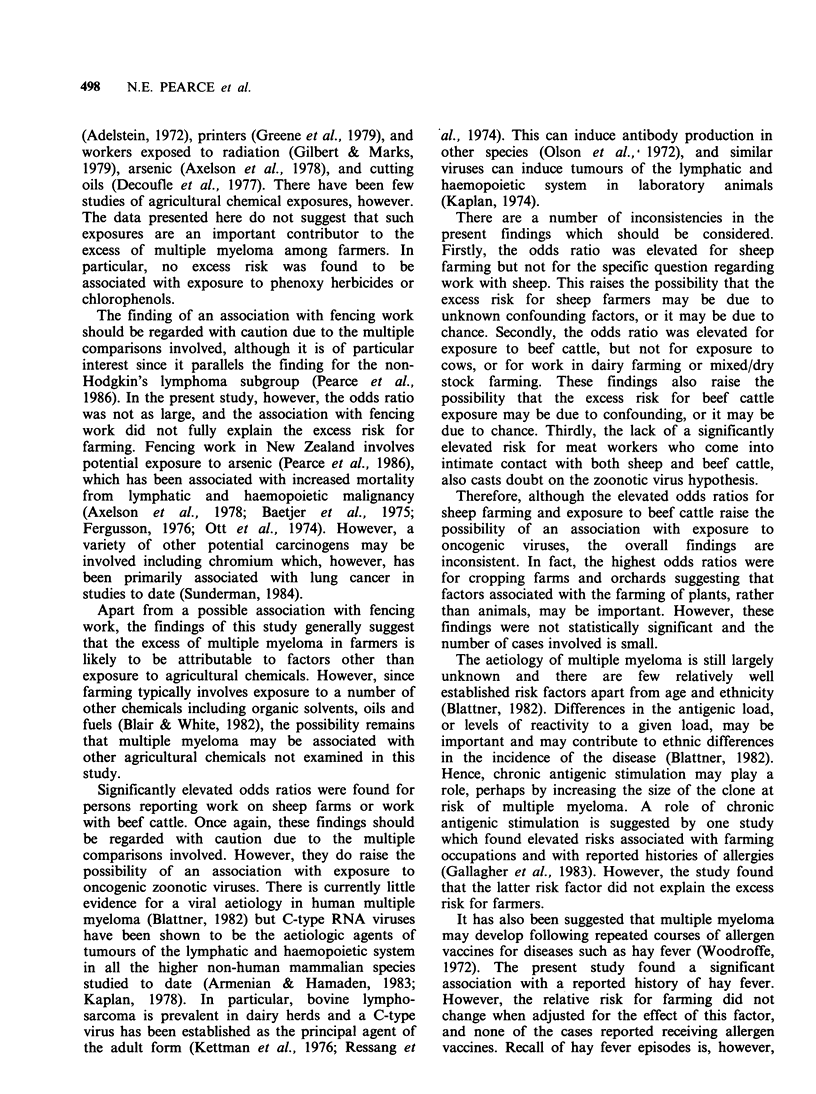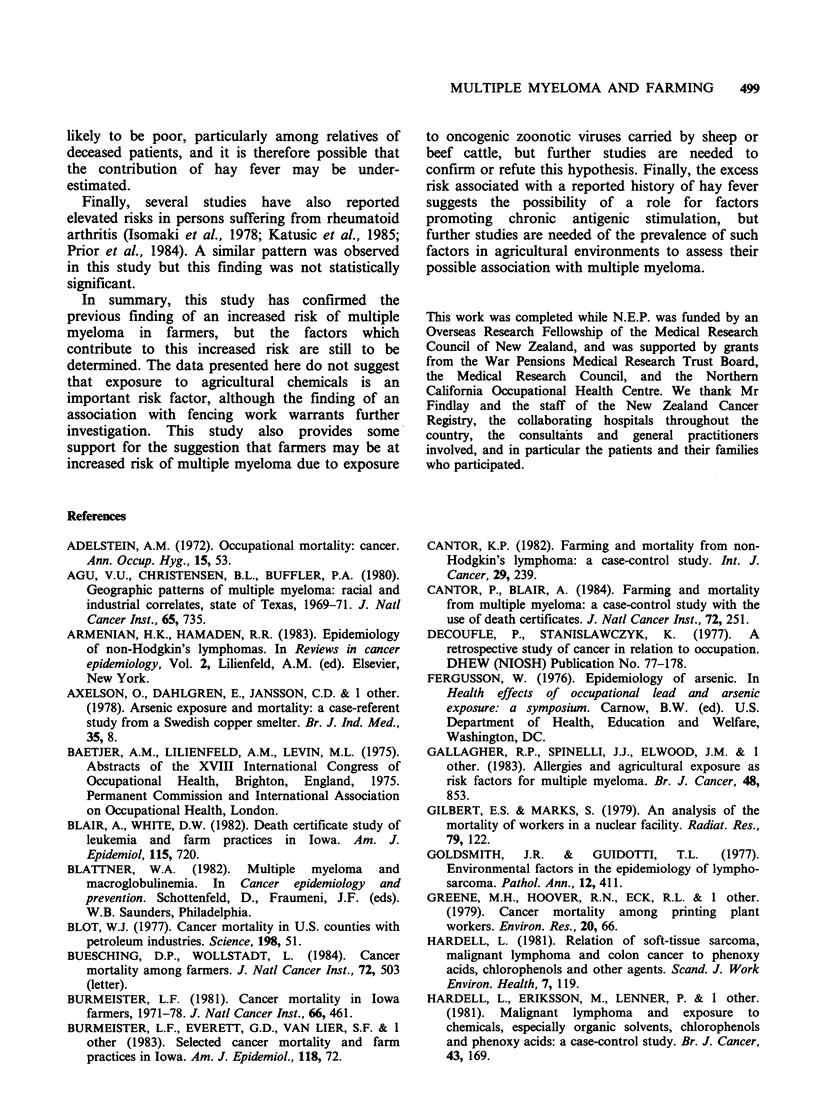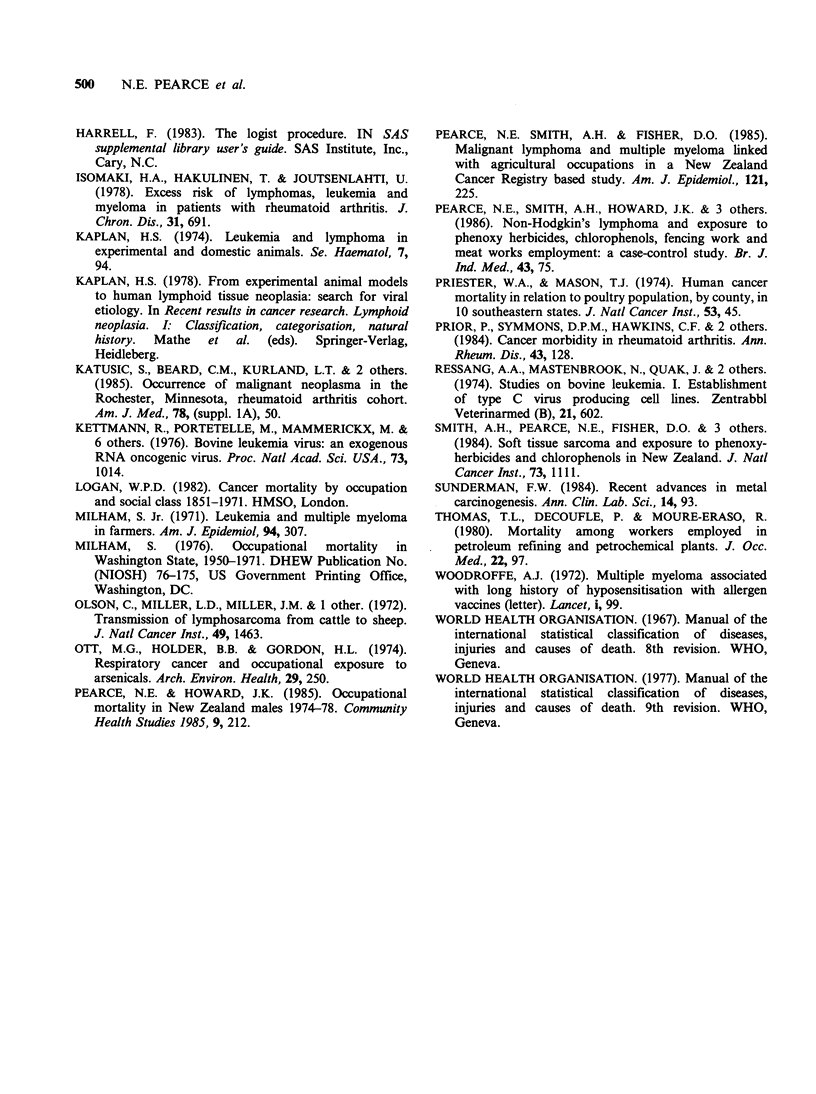# Case-control study of multiple myeloma and farming.

**DOI:** 10.1038/bjc.1986.202

**Published:** 1986-09

**Authors:** N. E. Pearce, A. H. Smith, J. K. Howard, R. A. Sheppard, H. J. Giles, C. A. Teague

## Abstract

A previous case-control study which utilised the occupational information available on the New Zealand Cancer Registry found an increased risk of multiple myeloma in agricultural workers consistent with previous findings in the United States. The findings are now presented for the second phase of the study which involved interviewing 76 cases of multiple myeloma (who had been included in the previous study) together with 315 controls with other types of cancer. The previous finding on an excess of farmers in the case group was confirmed by the interview data (odds ratio = 1.7, 95% confidence limits 1.0-2.9, P = 0.04). There were no significant differences between cases and controls regarding potential exposure to phenoxy herbicides or chlorophenols. There were also no significant differences regarding activities involving potential exposure to other agricultural chemicals, although the odds ratio for fencing work, which may involve exposure to arsenic and sodium pentachlorophenate, was 1.6 (95% confidence limits 0.9-2.7, P = 0.11). The odds ratios were significantly elevated for sheep farming (odds ratio = 1.9, 95% confidence limits 1.0-3.6, P = 0.04) and exposure to beef cattle (odds ratio = 1.7, 95% confidence limits 1.0-2.9, P = 0.05). The odds ratio was also elevated for persons reporting a history of hay fever (odds ratio = 1.9, 95% confidence limits 1.0-3.5, P = 0.05). Overall, these findings suggest that the search for the causes of elevated mortality in farmers from multiple myeloma should be directed to potential causes other than pesticide exposure.


*Br. J. Cancer* (1986), **54**, 493–500

### Case-control study of multiple myeloma and farming

N.E. Pearce[1,2], A.H. Smith[3], J.K. Howard[1], R.A. Sheppard[1], H.J. Giles[1] & C. A. Teague[4]

[1]*Department of Community Health, Wellington Clinical School of Medicine, Wellington Hospital, Wellington, New Zealand;* [2]*Department of Epidemiology, School of Public Health, University of North Carolina, Chapel Hill, NC 27514, USA;* [3]*Department of Biomedical and Environmental Health Sciences, School of Public Health, University of California, Berkeley, CA 94720, USA;* [4]*Department of Pathology, Princess Alexandra Hospital, Brisbane, Queensland 4102, Australia.*

**Summary** A previous case-control study which utilised the occupational information available on the New Zealand Cancer Registry found an increased risk of multiple myeloma in agricultural workers consistent with previous findings in the United States. The findings are now presented for the second phase of the study which involved interviewing 76 cases of multiple myeloma (who had been included in the previous study) together with 315 controls with other types of cancer. The previous finding on an excess of farmers in the case group was confirmed by the interview data (odds ratio = 1.7, 95% confidence limits 1.0–2.9, $P=0.04$). There were no significant differences between cases and controls regarding potential exposure to phenoxy herbicides or chlorophenols. There were also no significant differences regarding activities involving potential exposure to other agricultural chemicals, although the odds ratio for fencing work, which may involve exposure to arsenic and sodium pentachlorophenate, was 1.6 (95% confidence limits 0.9–2.7, $P=0.11$). The odds ratios were significantly elevated for sheep farming (odds ratio = 1.9, 95% confidence limits 1.0–3.6, $P=0.04$) and exposure to beef cattle (odds ratio = 1.7, 95% conficence limits 1.0–2.9, $P=0.05$). The odds ratio was also elevated for persons reporting a history of hay fever (odds ratio = 1.9, 95% confidence limits 1.0–3.5, $P=0.05$). Overall, these findings suggest that the search for the causes of elevated mortality in farmers from multiple myeloma should be directed to potential causes other than pesticide exposure.

A previous analysis of the occupations of male multiple myeloma and malignant lymphoma patients recorded on the New Zealand Cancer Registry during the period 1977–81, compared with other cancer controls, found that agricultural workers were at increased risk of developing malignant lymphoma and multiple myeloma (Pearce *et al.*, 1985). Similar excesses have been found in studies in other countries (Buesching & Wollstadt, 1984; Burmeister, 1981; Burmeister *et al.*, 1983; Canter, 1982; Cantor & Blair, 1984; Goldsmith & Guidotti, 1977; Logan, 1982), including Swedish studies which found an association between malignant lymphoma and exposure to phenoxy herbicides or chlorophenols (Hardell, 1981; Hardell *et al.*, 1981), both of which have been widely used in New Zealand since the late 1940's (Smith *et al.*, 1984).

The New Zealand excess was almost entirely among patients registered under codes 202 (non-Hodgkin's lymphoma other than lymphosarcoma and reticulosarcoma) and 203 (multiple myeloma) of the International Classification of Diseases (ICD) (WHO, 1967; 1977). Interview findings for the former subgroup have been presented previously (Pearce *et al.*, 1986), and demonstrated that the overall excess for farmers was attributable to excesses among farmers who had carried out fencing work or worked in a meat works, with particularly high risks for farmers who had carried out both activities. This paper presents the interview findings for the multiple myeloma subgroup.

Correspondence: N. Pearce
Received 10 March 1986; and in revised form, 24 April 1986.

### Methods

The study population comprised 102 male public hospital patients who were registered under ICD code 203 during the period 1977–81 and who were less than 70 years of age at time of registration.

For each of the cases, four male cancer patients who had the same year of registration and were within two years of birth had previously been chosen as controls for the study using Cancer Registry information on occupation (Pearce *et al.*, 1985). Two of the four control patients were selected at random for this study. Interviews were conducted by telephone by one interviewer who was not aware of whether the patient had a multiple myeloma or was a cancer control. The interviews were conducted concurrently with those of the other cancer controls for the non-Hodgkin's lymphoma subgroup (Pearce *et al.*, 1986), and the

© The Macmillan Press Ltd., 1986

two groups of controls have been pooled in the analysis presented here to provide greater precision for the estimates of control exposure. The latter study had also included a second control group chosen from the general population as an additional check which gave very similar findings to those obtained with the main control group of other cancer patients (Pearce et al., 1986).

The questionnaire was similar to that used in previously published case-control studies of soft tissue sarcoma (Smith et al., 1984) and non-Hodgkin's lymphoma (Pearce et al., 1986) patients. As before, stem questions were used to identify whether or not study subjects had worked in particular occupations in which there was potential for exposure to phenoxy herbicides or chlorophenols. If the response to a stem question was in the affirmative, then a series of subsidiary questions were asked to clarify the work done and the actual potential for exposure, firstly in general terms, and then in specific terms, seeking the identity of the chemicals used. Additional questions were also asked in this study concerning involvement in various types of farming, work with various farm animals, and history of various medical conditions and allergies. The medical conditions selected for questioning were those which had been associated with tumours of the lymphatic and haemopoietic system in epidemiologic studies or case-series reports, and which were expected to have sufficiently high incidence in the control population.

The Statistical Analysis System logist procedure (Harrell, 1983) was used to perform an unconditional maximum likelihood logistic regression analysis, adjusting for decade of birth and whether the subject or the next-of-kin was interviewed. Two-tailed *P*-values and 95% confidence limits were calculated for all analyses.

### Results

Relevant laboratory reports of cases coded as multiple myeloma were examined by one pathologist and five cases who did not appear to have multiple myeloma were excluded. Reasons for other exclusions included: private hospital patients who had been wrongly coded on the Cancer Registry; duplication on the Cancer Registry; wrongly coded age; and persons who had come to New Zealand solely for medical treatment. Interviews were completed with 76 (82%) of the 93 eligible cases and 315 (81%) of the 389 eligible controls.

Table I gives the findings for various occupations and activities potentially associated with exposure to phenoxy herbicides. Cases were more likely to have been farmers than controls, with the odds ratio of 1.7 being similar to that found using

**Table I** Odds ratio estimates for multiple myeloma for occupations and activities involving potential exposure to phenoxy herbicides[a].

| *Exposure* | *Exposed cases* | *Exposed controls* | *Odds ratio* | *95% Confidence limits* | *P value* |
|---|---|---|---|---|---|
| Farming | 43 | 113 | 1.7 | 1.0–2.9 | 0.04 |
| used any agricultural chemical spray | 16 | 53 | 1.3 | 0.7–2.5 | 0.37 |
| sprayed gorse, blackberry, pasture, cereal or peas | 14 | 40 | 1.6 | 0.8–3.1 | 0.18 |
| Forestry worker | 7 | 30 | 1.0 | 0.4–2.3 | 0.92 |
| sprayed chemicals | 0 | 1 | — | — | — |
| Railways worker | 11 | 36 | 1.3 | 0.6–2.7 | 0.51 |
| sprayed chemicals | 0 | 1 | — | — | — |
| Ministry of works | 1 | 26 | 0.1 | 0.0–1.1 | 0.06 |
| sprayed chemicals | 0 | 1 | — | — | — |
| Town council worker | 10 | 40 | 1.0 | 0.5–2.1 | 0.98 |
| sprayed chemicals | 1 | 10 | 0.4 | 0.0–3.2 | 0.39 |
| Chemical sprayer | 0 | 1 | — | — | — |
| Aerial spray work | 0 | 0 | — | — | — |

[a]Stratified on decade of birth and whether the subject or a relative was interviewed.

**Table II** Odds ratio estimates for multiple myeloma for various categories of exposure to phenoxy herbicides[a].

| *Exposure* | *Exposed cases* | *Exposed controls* | *Odds ratio* | *95% Confidence limits* | *P value* |
|---|---|---|---|---|---|
| Ever sprayed an agricultural chemical | 43 | 158 | 1.3 | 0.8–2.2 | 0.30 |
| Ever potentially exposed before cancer registration | 17 | 67 | 1.1 | 0.6–2.0 | 0.74 |
| Potential exposure of more than 1 day at least 5 years before cancer registration | 16 | 52 | 1.4 | 0.7–2.7 | 0.29 |
| Probable or definite exposure of more than 1 day at least 5 years before cancer registration | 13 | 46 | 1.3 | 0.6–2.5 | 0.48 |
| Probable or definite exposure of at least 5 days more than 10 years before cancer registration | 12 | 40 | 1.4 | 0.7–2.2 | 0.40 |

[a]Stratified on decade of birth and whether the subject or a relative was interviewed.

**Table III** Odds ratio estimates for multiple myeloma for occupations and activities involving potential exposure to chlorophenols[a].

| *Exposure* | *Exposed cases* | *Exposed controls* | *Odds ratio* | *95% Confidence limits* | *P value* |
|---|---|---|---|---|---|
| Fencing work | 29 | 87 | 1.6 | 0.9–2.7 | 0.11 |
| treated own posts | 2 | 8 | 1.1 | 0.2–5.6 | 0.87 |
| Saw mill or timber merchant | 11 | 42 | 1.1 | 0.5–2.3 | 0.81 |
| potential exposure at saw mill or timber merchant | 5 | 16 | 1.4 | 0.5–3.9 | 0.57 |
| Meat works | 15 | 49 | 1.3 | 0.7–2.5 | 0.39 |
| pelt department in meat works | 2 | 10 | 0.8 | 0.2–3.8 | 0.79 |
| Tannery | 1 | 7 | 0.6 | 0.1–5.1 | 0.65 |

[a]Stratified on decade of birth and whether the subject or a relative was interviewed.

Cancer Registry data (Pearce *et al.*, 1985). The odds ratios for spraying as a farmer were not significantly elevated and the proportions of cases and controls who had worked in other occupations which may involve spraying were also not significantly different.

Table II gives the specific findings relating to phenoxy herbicide exposure. None of the odds ratios was significantly elevated and the highest observed odds ratio – for the category of 'any agricultural chemical exposure' – was equal to that for farming in general (Table I).

Table III gives the findings for various occupations and activities potentially associated with exposure to chlorophenols while Table IV gives the specific findings relating to exposure. The

**Table IV** Odds ratio estimates for multiple myeloma for various categories of exposure to chlorophenols[a].

| *Exposure* | *Exposed cases* | *Exposed controls* | *Odds ratio* | *95% Confidence limits* | *P value* |
|---|---|---|---|---|---|
| Ever potentially exposed | 6 | 26 | 1.1 | 0.4–2.7 | 0.91 |
| Potential exposure of more than 1 day at least 5 years before cancer registration | 6 | 26 | 1.1 | 0.4–2.7 | 0.91 |
| Potential exposure of at least 5 days more than 10 years before cancer registration | 4 | 21 | 0.8 | 0.3–2.5 | 0.71 |

[a]Stratified on decade of birth and whether the subject or a relative was interviewed.

**Table V** Odds ratio estimates for multiple myeloma for various types of farming[a].

| *Exposure* | *Exposed cases* | *Exposed controls* | *Odds ratio* | *95% Confidence limits* | *P value* |
|---|---|---|---|---|---|
| Sheep farm | 18 | 43 | 1.9 | 1.0–3.6 | 0.04 |
| Dairy farm | 23 | 71 | 1.4 | 0.8–2.5 | 0.23 |
| Mixed/dry stock farm | 11 | 37 | 1.3 | 0.6–2.6 | 0.55 |
| Cropping farm | 5 | 10 | 2.0 | 0.6–6.0 | 0.24 |
| Poultry farm | 1 | 4 | 0.9 | 0.1–8.4 | 0.94 |
| Orchard | 2 | 3 | 2.8 | 0.5–16.9 | 0.27 |

[a]Stratified on decade of birth and whether the subject or a relative was interviewed.

elevated odds ratio for fencing work is of particular interest because it parallels the finding for the non-Hodgkin's lymphoma subgroup (Pearce *et al.*, 1986). However, the elevated risk associated with fencing work only partly accounted for the overall risk for farmers and the odds ratio for farmers who were not involved in fencing work was also elevated (odds ratio = 1.5, 95% confidence limits 0.8–3.1, $P = 0.24$).

Table V gives the findings for various types of farming work. There are few New Zealand farms solely involving beef cattle, but there are a number of 'mixed/dry stock' farms with both sheep and beef cattle. Cropping farms primarily involve market gardens which grow vegetables and fruit for the consumer market, but some involve the production of wheat and other grains. The odds ratios were elevated for work on sheep farms, cropping farms and orchards but only the former finding was statistically significant. The odds ratio for farmers who were not involved in sheep farming was 1.5 (95% confidence limits 0.8–2.6, $P = 0.20$).

Table VI gives the findings for reported exposure to various farm animals. The only significantly elevated odds ratio was for exposure to beef cattle.

Table VII gives the findings for various medical conditions and allergies. The only significantly elevated odds ratio was for persons reporting a history of hay fever. However, the odds ratio for farming did not change when adjusted for the effect of this factor.

### Discussion

The most important finding of this study is that farmers are at increased risk of multiple myeloma. This supports the similar findings from studies in other countries (Agu *et al.*, 1980; Burmeister, 1981; Burmeister *et al.*, 1983; Cantor & Blair, 1984; Gallagher *et al.*, 1983; Milham, 1971; Priester & Mason, 1974). It could be argued that the *P* value for farming is not valid, since this is not an independent analysis, but a further analysis (using

**Table VI** Odds ratio estimates for multiple myeloma for exposure to various farm animals[a].

| *Exposure* | *Exposed cases* | *Exposed controls* | *Odds ratio* | *95% Confidence limits* | *P value* |
|---|---|---|---|---|---|
| Sheep | 34 | 113 | 1.4 | 0.9–2.4 | 0.18 |
| Cows | 36 | 127 | 1.3 | 0.9–2.1 | 0.33 |
| Beef cattle | 31 | 91 | 1.7 | 1.0–2.9 | 0.05 |
| Poultry | 29 | 101 | 1.3 | 0.8–2.1 | 0.37 |
| Pigs | 27 | 92 | 1.3 | 0.8–2.3 | 0.32 |

[a]Stratified on decade of birth and whether the subject or a relative was interviewed.

**Table VII** Odds ratio estimates for multiple myeloma for various medical conditions and allergies[a].

| *Exposure* | *Exposed cases* | *Exposed controls* | *Odds ratio* | *95% Confidence limits* | *P value* |
|---|---|---|---|---|---|
| Rheumatoid arthritis | 4 | 7 | 2.3 | 0.6–8.0 | 0.21 |
| Eczema | 4 | 18 | 0.9 | 0.3–2.8 | 0.89 |
| Asthma | 9 | 29 | 1.3 | 0.6–2.9 | 0.53 |
| Asthma medication | 8 | 19 | 1.8 | 0.7–4.3 | 0.21 |
| Hay fever | 19 | 48 | 1.9 | 1.0–3.5 | 0.05 |
| Allergen vaccines | 0 | 5 | — | — | — |
| Food allergies | 3 | 6 | 2.1 | 0.5–8.5 | 0.32 |
| Drug allergies | 4 | 19 | 0.8 | 0.3–2.5 | 0.72 |

[a]Stratified on decade of birth and whether the subject or a relative was interviewed.

interview data) of a previously published study (Pearce *et al.*, 1985). However, this problem only applies in the context of significance testing, and does not affect the confidence limits for the odds ratio. Furthermore, it only affects the *P* value for farming, and not those for other variables. Furthermore, it is of interest that the analysis presented here also confirms the previous New Zealand finding based on Cancer Registry data (Pearce *et al.*, 1985). This involved the use of relatively crude information since it was based on the occupation reported at the time of cancer registration and no information on prior employment was available.

The problem of multiple comparisons is also of concern, as there are approximately 40 comparisons presented. Four of these were 'statistically significant', whereas it could be expected that two would be by chance alone. Hence, the findings should be regarded with caution, although they are not completely exploratory, since it was prior knowledge from previously published studies which lead to their consideration in this study.

A further methodological issue relates to the use of other cancers as controls. For example, if smoking was less common in farmers than elsewhere then they would be under-represented among other cancer registrants, and the odds ratio would be biased upwards. However, the previously published study (Pearce *et al.*, 1985), found that the proportion of farmers in the controls with cancers of the respiratory tract was actually slightly higher than in the rest of the control group. Furthermore, there are considerable advantages to using other cancers as controls since this minimises information bias, while any bias due to other cancers being associated with farming is likely to be small, since the overall cancer mortality in New Zealand farmers is identical to that for the general population (Pearce & Howard, 1985).

Preliminary surveys have suggested that multiple myeloma may occur excessively in petroleum refinery and petrochemical workers (Blot, 1977; Decoufle & Stanislawczyk, 1977; Thomas *et al.*, 1980), wood workers (Milham, 1976), leather workers (Decoufle *et al.*, 1977), food workers

(Adelstein, 1972), printers (Greene *et al.*, 1979), and workers exposed to radiation (Gilbert & Marks, 1979), arsenic (Axelson *et al.*, 1978), and cutting oils (Decoufle *et al.*, 1977). There have been few studies of agricultural chemical exposures, however. The data presented here do not suggest that such exposures are an important contributor to the excess of multiple myeloma among farmers. In particular, no excess risk was found to be associated with exposure to phenoxy herbicides or chlorophenols.

The finding of an association with fencing work should be regarded with caution due to the multiple comparisons involved, although it is of particular interest since it parallels the finding for the non-Hodgkin's lymphoma subgroup (Pearce *et al.*, 1986). In the present study, however, the odds ratio was not as large, and the association with fencing work did not fully explain the excess risk for farming. Fencing work in New Zealand involves potential exposure to arsenic (Pearce *et al.*, 1986), which has been associated with increased mortality from lymphatic and haemopoietic malignancy (Axelson *et al.*, 1978; Baetjer *et al.*, 1975; Fergusson, 1976; Ott *et al.*, 1974). However, a variety of other potential carcinogens may be involved including chromium which, however, has been primarily associated with lung cancer in studies to date (Sunderman, 1984).

Apart from a possible association with fencing work, the findings of this study generally suggest that the excess of multiple myeloma in farmers is likely to be attributable to factors other than exposure to agricultural chemicals. However, since farming typically involves exposure to a number of other chemicals including organic solvents, oils and fuels (Blair & White, 1982), the possibility remains that multiple myeloma may be associated with other agricultural chemicals not examined in this study.

Significantly elevated odds ratios were found for persons reporting work on sheep farms or work with beef cattle. Once again, these findings should be regarded with caution due to the multiple comparisons involved. However, they do raise the possibility of an association with exposure to oncogenic zoonotic viruses. There is currently little evidence for a viral aetiology in human multiple myeloma (Blattner, 1982) but C-type RNA viruses have been shown to be the aetiologic agents of tumours of the lymphatic and haemopoietic system in all the higher non-human mammalian species studied to date (Armenian & Hamaden, 1983; Kaplan, 1978). In particular, bovine lymphosarcoma is prevalent in dairy herds and a C-type virus has been established as the principal agent of the adult form (Kettman *et al.*, 1976; Ressang *et al.*, 1974). This can induce antibody production in other species (Olson *et al.*, 1972), and similar viruses can induce tumours of the lymphatic and haemopoietic system in laboratory animals (Kaplan, 1974).

There are a number of inconsistencies in the present findings which should be considered. Firstly, the odds ratio was elevated for sheep farming but not for the specific question regarding work with sheep. This raises the possibility that the excess risk for sheep farmers may be due to unknown confounding factors, or it may be due to chance. Secondly, the odds ratio was elevated for exposure to beef cattle, but not for exposure to cows, or for work in dairy farming or mixed/dry stock farming. These findings also raise the possibility that the excess risk for beef cattle exposure may be due to confounding, or it may be due to chance. Thirdly, the lack of a significantly elevated risk for meat workers who come into intimate contact with both sheep and beef cattle, also casts doubt on the zoonotic virus hypothesis.

Therefore, although the elevated odds ratios for sheep farming and exposure to beef cattle raise the possibility of an association with exposure to oncogenic viruses, the overall findings are inconsistent. In fact, the highest odds ratios were for cropping farms and orchards suggesting that factors associated with the farming of plants, rather than animals, may be important. However, these findings were not statistically significant and the number of cases involved is small.

The aetiology of multiple myeloma is still largely unknown and there are few relatively well established risk factors apart from age and ethnicity (Blattner, 1982). Differences in the antigenic load, or levels of reactivity to a given load, may be important and may contribute to ethnic differences in the incidence of the disease (Blattner, 1982). Hence, chronic antigenic stimulation may play a role, perhaps by increasing the size of the clone at risk of multiple myeloma. A role of chronic antigenic stimulation is suggested by one study which found elevated risks associated with farming occupations and with reported histories of allergies (Gallagher *et al.*, 1983). However, the study found that the latter risk factor did not explain the excess risk for farmers.

It has also been suggested that multiple myeloma may develop following repeated courses of allergen vaccines for diseases such as hay fever (Woodroffe, 1972). The present study found a significant association with a reported history of hay fever. However, the relative risk for farming did not change when adjusted for the effect of this factor, and none of the cases reported receiving allergen vaccines. Recall of hay fever episodes is, however, likely to be poor, particularly among relatives of deceased patients, and it is therefore possible that the contribution of hay fever may be underestimated.

Finally, several studies have also reported elevated risks in persons suffering from rheumatoid arthritis (Isomaki *et al.*, 1978; Katusic *et al.*, 1985; Prior *et al.*, 1984). A similar pattern was observed in this study but this finding was not statistically significant.

In summary, this study has confirmed the previous finding of an increased risk of multiple myeloma in farmers, but the factors which contribute to this increased risk are still to be determined. The data presented here do not suggest that exposure to agricultural chemicals is an important risk factor, although the finding of an association with fencing work warrants further investigation. This study also provides some support for the suggestion that farmers may be at increased risk of multiple myeloma due to exposure to oncogenic zoonotic viruses carried by sheep or beef cattle, but further studies are needed to confirm or refute this hypothesis. Finally, the excess risk associated with a reported history of hay fever suggests the possibility of a role for factors promoting chronic antigenic stimulation, but further studies are needed of the prevalence of such factors in agricultural environments to assess their possible association with multiple myeloma.

This work was completed while N.E.P. was funded by an Overseas Research Fellowship of the Medical Research Council of New Zealand, and was supported by grants from the War Pensions Medical Research Trust Board, the Medical Research Council, and the Northern California Occupational Health Centre. We thank Mr Findlay and the staff of the New Zealand Cancer Registry, the collaborating hospitals throughout the country, the consultants and general practitioners involved, and in particular the patients and their families who participated.

### References

ADELSTEIN, A.M. (1972). Occupational mortality: cancer. *Ann. Occup. Hyg.*, **15**, 53.

AGU, V.U., CHRISTENSEN, B.L., BUFFLER, P.A. (1980). Geographic patterns of multiple myeloma: racial and industrial correlates, state of Texas, 1969–71. *J. Natl Cancer Inst.*, **65**, 735.

ARMENIAN, H.K., HAMADEN, R.R. (1983). Epidemiology of non-Hodgkin's lymphomas. In *Reviews in cancer epidemiology*, Vol. **2**, Lilienfeld, A.M. (ed). Elsevier, New York.

AXELSON, O., DAHLGREN, E., JANSSON, C.D. & 1 other. (1978). Arsenic exposure and mortality: a case-referent study from a Swedish copper smelter. *Br. J. Ind. Med.*, **35**, 8.

BAETJER, A.M., LILIENFELD, A.M., LEVIN, M.L. (1975). Abstracts of the XVIII International Congress of Occupational Health, Brighton, England, 1975. Permanent Commission and International Association on Occupational Health, London.

BLAIR, A., WHITE, D.W. (1982). Death certificate study of leukemia and farm practices in Iowa. *Am. J. Epidemiol*, **115**, 720.

BLATTNER, W.A. (1982). Multiple myeloma and macroglobulinemia. In *Cancer epidemiology and prevention*. Schottenfeld, D., Fraumeni, J.F. (eds). W.B. Saunders, Philadelphia.

BLOT, W.J. (1977). Cancer mortality in U.S. counties with petroleum industries. *Science*, **198**, 51.

BUESCHING, D.P., WOLLSTADT, L. (1984). Cancer mortality among farmers. *J. Natl Cancer Inst.*, **72**, 503 (letter).

BURMEISTER, L.F. (1981). Cancer mortality in Iowa farmers, 1971–78. *J. Natl Cancer Inst.*, **66**, 461.

BURMEISTER, L.F., EVERETT, G.D., VAN LIER, S.F. & 1 other (1983). Selected cancer mortality and farm practices in Iowa. *Am. J. Epidemiol.*, **118**, 72.

CANTOR, K.P. (1982). Farming and mortality from non-Hodgkin's lymphoma: a case-control study. *Int. J. Cancer*, **29**, 239.

CANTOR, P., BLAIR, A. (1984). Farming and mortality from multiple myeloma: a case-control study with the use of death certificates. *J. Natl Cancer Inst.*, **72**, 251.

DECOUFLE, P., STANISLAWCZYK, K. (1977). A retrospective study of cancer in relation to occupation. DHEW (NIOSH) Publication No. 77–178.

FERGUSSON, W. (1976). Epidemiology of arsenic. In *Health effects of occupational lead and arsenic exposure: a symposium*. Carnow, B.W. (ed). U.S. Department of Health, Education and Welfare, Washington, DC.

GALLAGHER, R.P., SPINELLI, J.J., ELWOOD, J.M. & 1 other. (1983). Allergies and agricultural exposure as risk factors for multiple myeloma. *Br. J. Cancer*, **48**, 853.

GILBERT, E.S. & MARKS, S. (1979). An analysis of the mortality of workers in a nuclear facility. *Radiat. Res.*, **79**, 122.

GOLDSMITH, J.R. & GUIDOTTI, T.L. (1977). Environmental factors in the epidemiology of lymphosarcoma. *Pathol. Ann.*, **12**, 411.

GREENE, M.H., HOOVER, R.N., ECK, R.L. & 1 other. (1979). Cancer mortality among printing plant workers. *Environ. Res.*, **20**, 66.

HARDELL, L. (1981). Relation of soft-tissue sarcoma, malignant lymphoma and colon cancer to phenoxy acids, chlorophenols and other agents. *Scand. J. Work Environ. Health*, **7**, 119.

HARDELL, L., ERIKSSON, M., LENNER, P. & 1 other. (1981). Malignant lymphoma and exposure to chemicals, especially organic solvents, chlorophenols and phenoxy acids: a case-control study. *Br. J. Cancer*, **43**, 169.

HARRELL, F. (1983). The logist procedure. IN *SAS supplemental library user's guide*. SAS Institute, Inc., Cary, N.C.

ISOMAKI, H.A., HAKULINEN, T. & JOUTSENLAHTI, U. (1978). Excess risk of lymphomas, leukemia and myeloma in patients with rheumatoid arthritis. *J. Chron. Dis.*, **31**, 691.

KAPLAN, H.S. (1974). Leukemia and lymphoma in experimental and domestic animals. *Se. Haematol*, **7**, 94.

KAPLAN, H.S. (1978). From experimental animal models to human lymphoid tissue neoplasia: search for viral etiology. In *Recent results in cancer research. Lymphoid neoplasia. I: Classification, categorisation, natural history*. Mathe *et al.* (eds). Springer-Verlag, Heidleberg.

KATUSIC, S., BEARD, C.M., KURLAND, L.T. & 2 others. (1985). Occurrence of malignant neoplasma in the Rochester, Minnesota, rheumatoid arthritis cohort. *Am. J. Med.*, **78**, (suppl. 1A), 50.

KETTMANN, R., PORTETELLE, M., MAMMERICKX, M. & 6 others. (1976). Bovine leukemia virus: an exogenous RNA oncogenic virus. *Proc. Natl Acad. Sci. USA.*, **73**, 1014.

LOGAN, W.P.D. (1982). Cancer mortality by occupation and social class 1851–1971. HMSO, London.

MILHAM, S. Jr. (1971). Leukemia and multiple myeloma in farmers. *Am. J. Epidemiol*, **94**, 307.

MILHAM, S. (1976). Occupational mortality in Washington State, 1950–1971. DHEW Publication No. (NIOSH) 76–175, US Government Printing Office, Washington, DC.

OLSON, C., MILLER, L.D., MILLER, J.M. & 1 other. (1972). Transmission of lymphosarcoma from cattle to sheep. *J. Natl Cancer Inst.*, **49**, 1463.

OTT, M.G., HOLDER, B.B. & GORDON, H.L. (1974). Respiratory cancer and occupational exposure to arsenicals. *Arch. Environ. Health*, **29**, 250.

PEARCE, N.E. & HOWARD, J.K. (1985). Occupational mortality in New Zealand males 1974–78. *Community Health Studies 1985*, **9**, 212.

PEARCE, N.E. SMITH, A.H. & FISHER, D.O. (1985). Malignant lymphoma and multiple myeloma linked with agricultural occupations in a New Zealand Cancer Registry based study. *Am. J. Epidemiol.*, **121**, 225.

PEARCE, N.E., SMITH, A.H., HOWARD, J.K. & 3 others. (1986). Non-Hodgkin's lymphoma and exposure to phenoxy herbicides, chlorophenols, fencing work and meat works employment: a case-control study. *Br. J. Ind. Med.*, **43**, 75.

PRIESTER, W.A., & MASON, T.J. (1974). Human cancer mortality in relation to poultry population, by county, in 10 southeastern states. *J. Natl Cancer Inst.*, **53**, 45.

PRIOR, P., SYMMONS, D.P.M., HAWKINS, C.F. & 2 others. (1984). Cancer morbidity in rheumatoid arthritis. *Ann. Rheum. Dis.*, **43**, 128.

RESSANG, A.A., MASTENBROOK, N., QUAK, J. & 2 others. (1974). Studies on bovine leukemia. I. Establishment of type C virus producing cell lines. Zentrabbl Veterinarmed (B), **21**, 602.

SMITH, A.H., PEARCE, N.E., FISHER, D.O. & 3 others. (1984). Soft tissue sarcoma and exposure to phenoxy-herbicides and chlorophenols in New Zealand. *J. Natl Cancer Inst.*, **73**, 1111.

SUNDERMAN, F.W. (1984). Recent advances in metal carcinogenesis. *Ann. Clin. Lab. Sci.*, **14**, 93.

THOMAS, T.L., DECOUFLE, P. & MOURE-ERASO, R. (1980). Mortality among workers employed in petroleum refining and petrochemical plants. *J. Occ. Med.*, **22**, 97.

WOODROFFE, A.J. (1972). Multiple myeloma associated with long history of hyposensitisation with allergen vaccines (letter). *Lancet*, **i**, 99.

WORLD HEALTH ORGANISATION. (1967). Manual of the international statistical classification of diseases, injuries and causes of death. 8th revision. WHO, Geneva.

WORLD HEALTH ORGANISATION. (1977). Manual of the international statistical classification of diseases, injuries and causes of death. 9th revision. WHO, Geneva.